# Heat shock protein 90 inhibitors augment endogenous wild-type p53 expression but down-regulate the adenovirally-induced expression by inhibiting a proteasome activity

**DOI:** 10.18632/oncotarget.25452

**Published:** 2018-05-25

**Authors:** Kuan Chai, Xuerao Ning, Thảo Thi Thanh Nguyễn, Boya Zhong, Takao Morinaga, Zhihan Li, Masato Shingyoji, Yuji Tada, Koichiro Tatsumi, Hideaki Shimada, Kenzo Hiroshima, Naoto Yamaguchi, Masatoshi Tagawa

**Affiliations:** ^1^ Division of Pathology and Cell Therapy, Chiba Cancer Center Research Institute, Chuo-ku, Chiba 260-8717, Japan; ^2^ Laboratory of Molecular Cell Biology, Graduate School of Pharmaceutical Sciences, Chiba University, Chuo-ku, Chiba 260-8675, Japan; ^3^ Department of Molecular Biology and Oncology, Graduate School of Medicine, Chiba University, Chuo-ku, Chiba 260-8670, Japan; ^4^ Division of Respirology, Chiba Cancer Center, Chuo-ku, Chiba 260-8717, Japan; ^5^ Department of Respirology, Graduate School of Medicine, Chiba University, Chuo-ku, Chiba 260-8670, Japan; ^6^ Department of Surgery, School of Medicine, Toho University, Tokyo 143-8540, Japan; ^7^ Department of Pathology, Tokyo Women's Medical University Yachiyo Medical Center, Yachiyo 276-8524, Japan

**Keywords:** adenovirus, p53, HSP90 inhibitor, proteasome

## Abstract

Heat shock protein 90 (HSP90) inhibitors suppressed MDM4 functions which mediated p53 ubiquitination, and blocked a chaperon function which influenced expression of the client proteins. We examined cytotoxic effects of the inhibitors, 17-allylamino-17-demetheoxygeldanamycin (17-AAG) and 17-dimethylaminoethylamino-17-demethoxy-geldanamycin (17-DMAG), on mesothelioma and investigated combinatory effects of the inhibitors and adenoviruses expressing the wild-type *p53* gene (Ad-p53). A majority of mesothelioma lacks p14 and p16 expression, which leads to defective p53 pathway despite bearing the wild-type p53 genotype. The HSP90 inhibitors up-regulated endogenous wild-type *p53* expression and induced cell death. Furthermore, the inhibitors increased the endogenous p53 levels that were induced by cisplatin. Nevertheless, the HSP90 inhibitors suppressed Ad-p53-induced exogenous p53 expression primarily at a posttranscriptional level and inhibited the Ad-p53-mediated cell death. HSP90 inhibitors suppressed ubiquitination processes which were involved in p53 degradation, but a proteasome inhibitor, MG-132, prevented the HSP90 inhibitors-induced p53 down-regulation. In contrast, an inhibitor for HSP70 with a chaperon function, pifithrin-μ, did not produce the p53 down-regulation. The HSP90 inhibitors did not suppress expression of Ad receptor molecules but rather increased expression of green fluorescence protein transduced by the same Ad vector. These data collectively indicated that an HSP90 inhibitor possessed a divalent action on p53 expression, as an activator for endogenous wild-type p53 through inhibited ubiquitination and a negative regulator of exogenously over-expressed p53 through the proteasome pathway.

## INTRODUCTION

Malignant mesothelioma develops mainly in the pleural cavity and disturbs functions of vital organs in the vicinity [1]. Chemotherapy is the primary treatment in most of the cases but the first-line regimen, combination of cisplatin and pemetrexed, remains unchanged for more than decades [2]. Genetic characterization of the clinical specimens showed that a majority of mesothelioma were defective of the INK4A/ARF locus, which contained the *p16^INK4A^* and the *p14^ARF^* genes, but possessed the wild-type *p53* gene [3]. Deletion of p16 expression increases cyclin-dependent kinase 4/6 activities and subsequently phosphorylates pRb, which induces uninhibited cell cycle progression. In addition, p14 deficiency augments MDM2 activities that promote p53 ubiquitination and degradation, and consequently decreases p53 expression levels. The genetic defect in the INK4A/ARF locus thus leads to dysfunction of both pRb and p53 with tumor suppressive activities. Up-regulation of p53 in mesothelioma not only restores the suppressed p53 functions but dephosphorylates pRb since p21 induced by p53 blocks cyclin-dependent kinase 2 activities. Induction of p53 expression is therefore a direct way to reconstitute the tumor suppressor functions and can be a therapeutic strategy for mesothelioma [4]. We in fact showed that transduction of mesothelioma with adenoviruses (Ad) expressing the wild-type *p53* gene (Ad-p53) decreased the viability and increased susceptibility to cisplatin- or pemetrexed-mediated cytotoxicity [5].

Heat shock protein (HSP) 90 is a molecular chaperon and functions to maintain a number of client proteins in order to deal with cellular stresses and to mediate cellular homeostasis [6]. The HSP90 expression is often up-regulated in a variety of cancer and the client proteins with oncogenic potential are therefore constitutively activated to support cancer cell survival. An inhibitor for HSP90 disrupts actions of the oncoproteins and produces cytotoxic effects on tumor cells which are frequently addicted to oncogenic processes [7]. The inhibitors also suppress growth signaling activities and have been investigated for anti-tumor effects in clinical trials [7, 8]. Moreover, HSP90 inhibitors can augment p53 expression through inhibiting functions of MDM4 which constitutes a heterodimeric structure with MDM2 [9, 10]. A degradation process of p53 is primarily mediated by the ubiquitination-proteasome pathway, and MDM2 with an ubiquitin ligase function negatively regulates p53 expression through facilitating the proteasome-mediated degradation [11]. HSP90 inhibitors therefore increase p53 expression by suppressing the MDM2-mediated p53 degradation through MDM4. The inhibitors can therefore be a candidate agent for therapy of mesothelioma which is sensitive to p53-mediated growth inhibition. Geldanamycin derivatives, 17-allylamino-17-demetheoxygeldanamycin (17-AAG) and 17-dimethylaminoethylamino-17-demethoxy-geldanamycin (17-DMAG), are a prototype of the HSP90 inhibitor but have not been well investigated for the cytotoxic activity in mesothelioma.

In this study we examined whether HSP90 inhibitors produced anti-tumor effects on mesothelioma and achieved combinatory effects with Ad-p53 by inhibiting a degradation process of transduced p53. We found that the HSP90 inhibitors augmented endogenous wild-type p53 expression but rather down-regulated the p53 level induced by Ad-p53.

## RESULTS

### Cytotoxic activity of HSP90 inhibitors to mesothelioma

**Figure 1:**
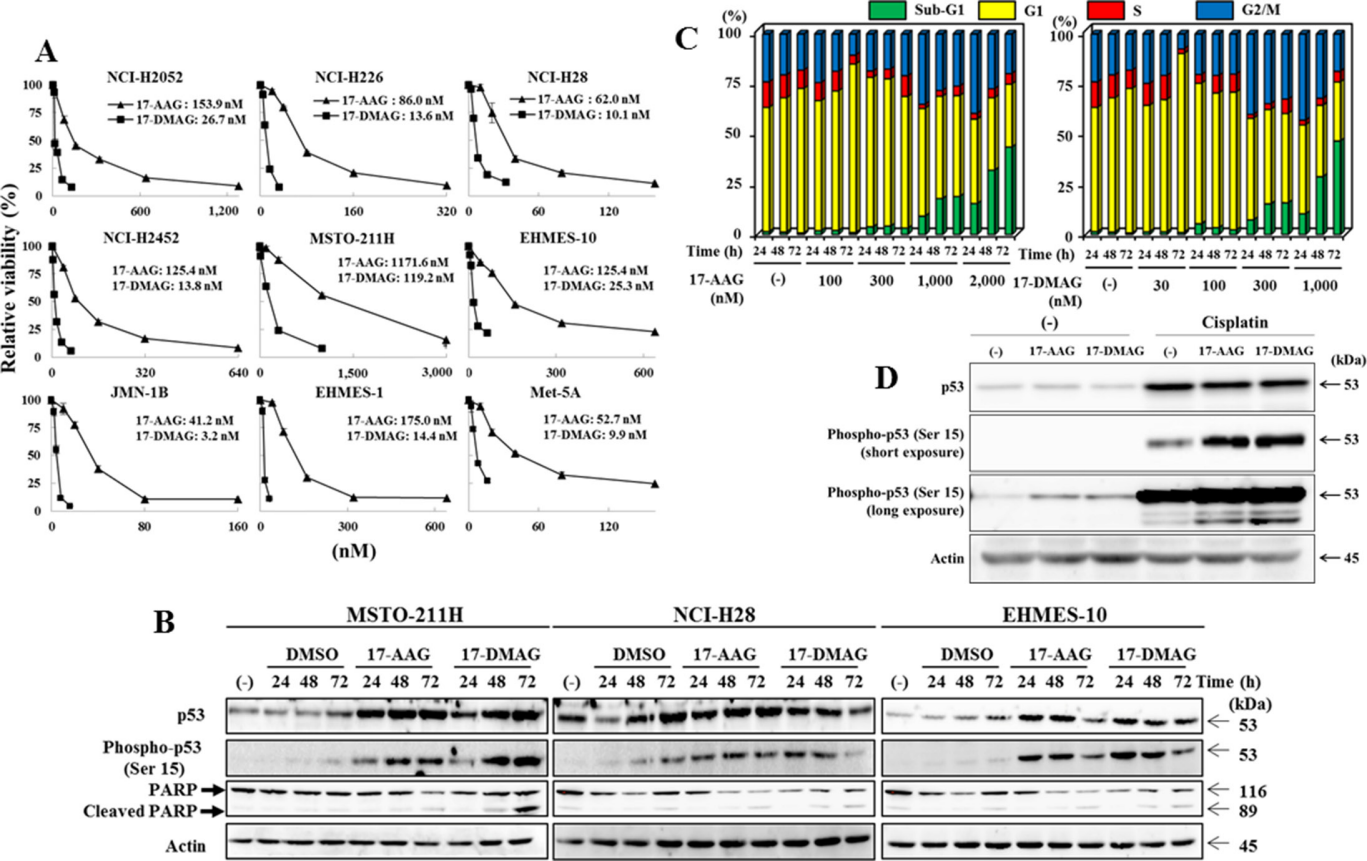
HSP90 inhibitors inhibited cell viability and increased p53 expression (**A**) Mesothelioma cells and immortalized Met-5A cells were treated with various doses of 17-AAG or 17-DMAG for 5 days, and the relative viability of was examined with the WST assay. The average with SE bars (*n* = 3) and IC_50_ values are shown. (**B**) MSTO-211H, NCI-H28 and EHMES-10 cells were treated with 17-AAG (2 μM), 17-DMAG (1 μM) or DMSO as a control as indicated. The cell lysate was subjected to Western blot analysis. Actin was used as a loading control. (**C**) Cell cycle progression of MSTO-211H cells treated with HSP90 inhibitors as indicated. The data showed an average of respective subpopulations in Supplementary Table 2. (**D**) NCI-H28 cells were treated with or without cisplatin (20 μM) and with 17-AAG (1 μM) or 17-DMAG (0.1 μM) for 48 hours. The cell lysate was subjected to Western blot analysis. Actin was used as a loading control.

We examined cytotoxic effects of 17-AAG and 17-DMAG with human mesothelioma cells and immortalized cells of mesothelium origin with the WST assay (Figure 1A). Relative viabilities of the cells were examined with different doses of the HSP90 inhibitors. The HSP90 inhibitors suppressed viability of these cells and 17-DMAG was more cytotoxic than 17-AAG. We then examined a possible relation between the susceptibility and the p53 functional status. We classified NCI-H2452 (truncated p53 protein), Met-5A (SV40 T antigen expressed), JMN-1B and EHMES-1 cells (mutated *p53* genotype) as a non-functional and other 5 cells as a functional p53 group. Comparison of a half maximal inhibitory concentration (IC_50_) values showed that the HSP90 inhibitors was in more effective to cells of the p53 non-functional type (IC_50_ values, 17-AAG average: 98.6 nM, 17-DMAG: 10.3 nM) than those of the p53 functional type (17-AAG: 319.8 nM, 17-DMAG: 39.0 nM) without statistical significance (17-AAG: *P* = 0.089, 17-DMAG: *P* = 0.086). We also examined a possible correlation between the susceptibility to HSP90 inhibitors and MDM4 expression levels (high MDM4: MSTO-211H, NCI-H28, EHMES-1, JMN-1B and Met-5A cells; low MDM4: NCI-H226, NCI-H2052, NCI-H2452 and EHMES-10 cells) (Supplementary Figure 1) and found it unrelated (17-AAG: *P* = 0.50, 17-DMAG: *P* = 0.66).

### HSP90 inhibitors augmented endogenous p53 expression and induced cell death

We examined whether HSP90 inhibitors increased p53 expression in the *p53* wild-type mesothelioma, MSTO-211H, NCI-H28 and EHMES-10 cells, which showed differential susceptibility to the HSP90 inhibitors. All the cells treated with HSP90 inhibitors up-regulated p53 expression and the phosphorylation at Ser 15 residue, a marker for p53 stabilization, and then showed cleavage of PARP (Figure 1B) (Summarized signal intensity of key molecules in each Western blot analysis in Supplementary Table 1). Cell cycle progression of MSTO-211H cells treated with HSP90 inhibitors showed that the treated cells increased G2/M at 24 hours and sub-G1 populations thereafter (Figure 1C, Supplementary Table 2). We also examined whether p53 expression induced by a DNA damaging agent, cisplatin, was further up-regulated with HSP90 inhibitors (Figure 1D). NCI-H28 cells with the wild-type *p53* genotype increased p53 expression with cisplatin, and HSP90 inhibitors augmented p53 phosphorylation not only in cisplatin-unstimulated but also -treated cells. These data collectively indicated that HSP90 inhibitors increased endogenous wild-type p53 levels and the up-regulation can be associated with cell death of mesothelioma with the wild-type *p53* genotype.

### HSP90 inhibitors suppressed Ad-p53-induced p53 expression

**Figure 2:**
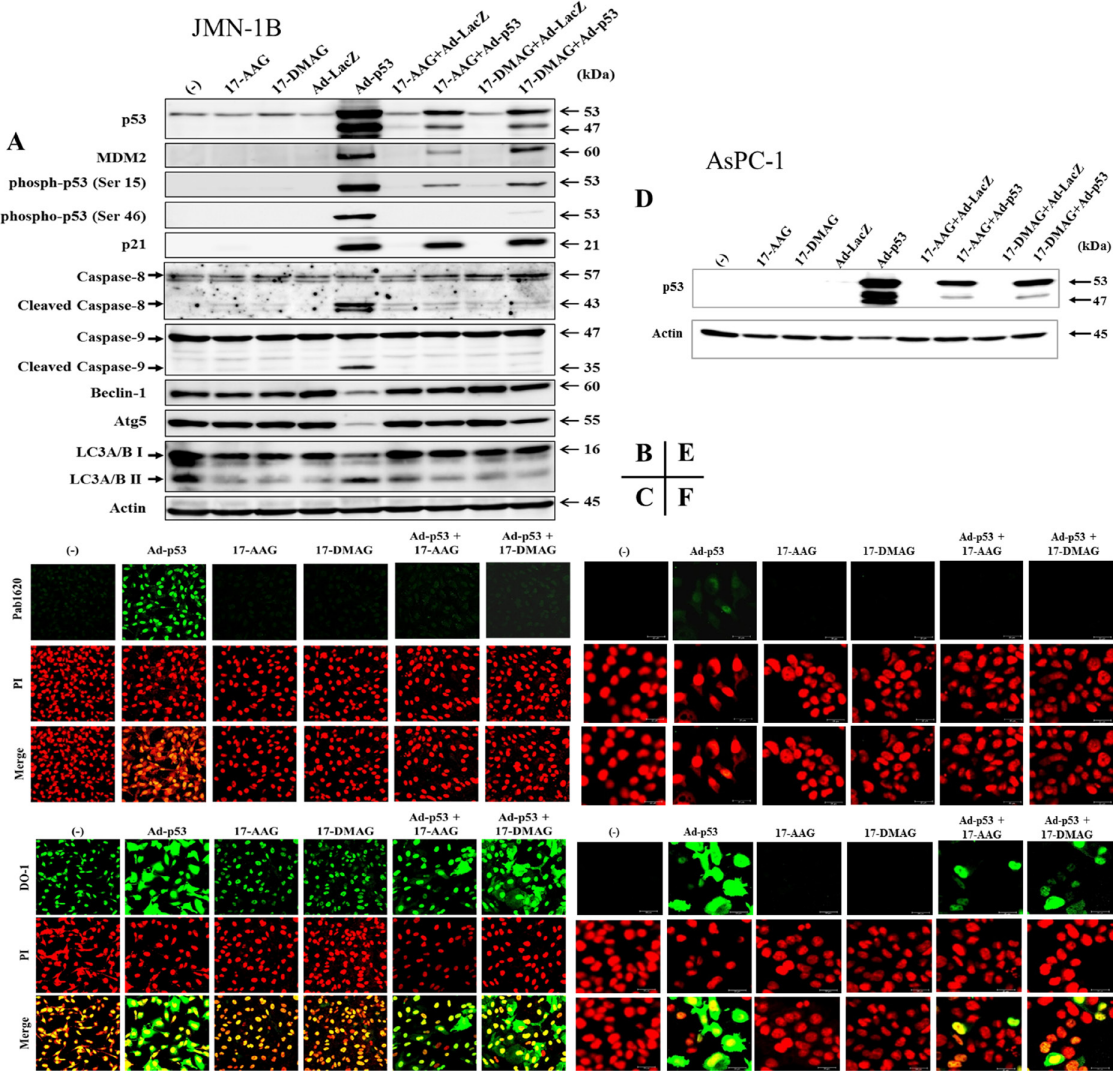
Expression of molecules involved in the p53 and autophagy pathways (**A**, **D**) JMN-1B (A) or AsPC-1 (D) cells were treated with 17-AAG (1 μM), 17-DMAG (0.1 μM), Ad-p53 (3 × 10^4^ vp/cell), Ad-LacZ (3 × 10^4^ vp/cell) or combination as indicated for 48 hours. JMN-1B cells expressed MDM2 predominantly at 60 kDa. Actin was used as loading control. (**B**, **C**, **E**, **F**) Representative immunofluorescence staining of JMN-1B (B, C) or AsPC-1 (E, F) cells with anti-p53 Ab under the same imaging condition. Cells treated with 17-AAG (1 μM) or 17-DMAG (0.1 μM), or with Ad-p53 (3 × 10^4^ vp/cell) together with 17-AAG (1 μM) or 17-DMAG (0.1 μM), were stained with anti-p53Ab, (B, E) Pab1620 or (C, F) DO-1, and PI.

We then examined influence of HSP90 inhibitors on p53 expression in JMN-1B cells with mutated *p53* genotype (Figure 2A). HSP90 inhibitors minimally affected the mutated p53 expression, and transduction with Ad-p53 but not with Ad expressing the *β-galactosidase* gene (Ad-LacZ) increased p53 levels. The majority of p53 in cells transduced with Ad-p53 was wild-type p53 and Western blot analysis detected additional p53 bands probably due to overproduction of exogenous p53. The transduction also induced p53 phosphorylation at Ser 15 and Ser 46 residues, and cleavages of caspase-8 and -9. Expression levels of p21 and MDM2, both of which were p53 target molecules, were up-regulated. These data indicated that the p53 downstream pathway leading to caspase cleavages was intact in JMN-1B cells. In contrast, transduction with Ad-p53 down-regulated Beclin-1, Atg5 and LC3A/B I expression but induced conversion from LC3A/B I to LC3A/B II. HSP90 inhibitors alone and Ad-LacZ did not influence Beclin-1 or Atg5 levels, but decreased both LC3A/BI and LC3A/B II expressions in comparison with untreated cells. These data suggested that Ad-p53 activated the apoptotic pathway through up-regulated p53 and could antagonize autophagy processes.

We further investigated effects of HSP90 inhibitors on the Ad-p53-mediated responses. The inhibitors rather decreased p53 expression and the phosphorylation levels induced by Ad-p53, and consequently the p53 downstream pathway was less activated. Cleavages of caspase-8 and -9 were down-regulated, and expression of MDM2 and to a less extent p21 decreased. Expression levels of autophagy-related molecules in cells treated with both Ad-p53 and HSP90 inhibitors were similar to those in cells treated with HSP90 inhibitors or Ad-LacZ alone. These data showed that HSP90 inhibitors produced negative effects on the Ad-p53-induced p53 expression.

JMN-1B cells infected with Ad-p53 expressed both p53 types, endogenous mutated p53 and exogenous wild-type p53. We discriminated the both type with 2 kinds of antibody (Ab), Pab1620 specifically reacting to wild-type p53 and DO-1 recognizing all the p53 types including mutated sequences. Immunofluorescence of JMN-1B cells with Pab1620 Ab detected wild-type p53 in Ad-p53-infected cells and showed that the p53 expression was down-regulated with HSP90 inhibitors (Figure 2B). In contrast, JMN-1B cells stained with DO-1 detected p53 in both cytoplasmic and nucleic regions in Ad-p53-infected cells and showed that HSP90 inhibitors decreased expression of exogenously transduced wild-type p53 in both cytoplasm and nucleus (Figure 2C, Supplementary Figure 2). HSP90 inhibitors did not influence on expression of mutated p53 under the experimental condition. These data indicated that HSP90 inhibitors suppressed expression of exogenously overexpressed wild-type p53 and had little effect on endogenous mutated p53.

We also used p53-null cells to exclude a possible effect of endogenous p53 on the wild-type p53 transduced. Mesothelioma cells with *p53*-null genotype were not available and we used pancreatic carcinoma, AsPC-1 cells, to examined the effects of HSP90 inhibitors on wild-type p53 (Figure 2D–2F). AsPC-1 cells transduced with Ad-p53 expressed wild-type p53 and the expression was down-regulated with HSP90 inhibitors (Figure 2D, Supplementary Figure 2). Immunostaining data showed that the transduced AsPC-1 cells became positive for both Pab1620 and DO-1, and the transduced cells treated with HSP90 inhibitors decreased the fluorescence intensity detected with both Ab (Figure 2E and 2F). These data collectively indicated that endogenous mutated p53 did not influence on Ad-p53- and HSP90 inhibitors-mediated effects. We noticed differential p53 staining profiles in AsPC-1 cells as well as in JMN-1B cells. Staining with Pab1620 detected p53 in nucleus and that with DO-1 detected p53 both in cytoplasm and nucleus, which presumably due to differential Ab affinity to p53. DO-1 has greater affinity than Pab1620, which was evidenced by staining of AsPC-1 cells transduced with Ad-p53. We presume that the cytoplasmic staining with DO-1 could be attributable to p53 detected during the biosynthesis or a leakage of fluorescence from stained nuclei with overexpressed p53.

### HSP90 inhibitors suppressed Ad-p53-mediated effects

**Figure 3:**
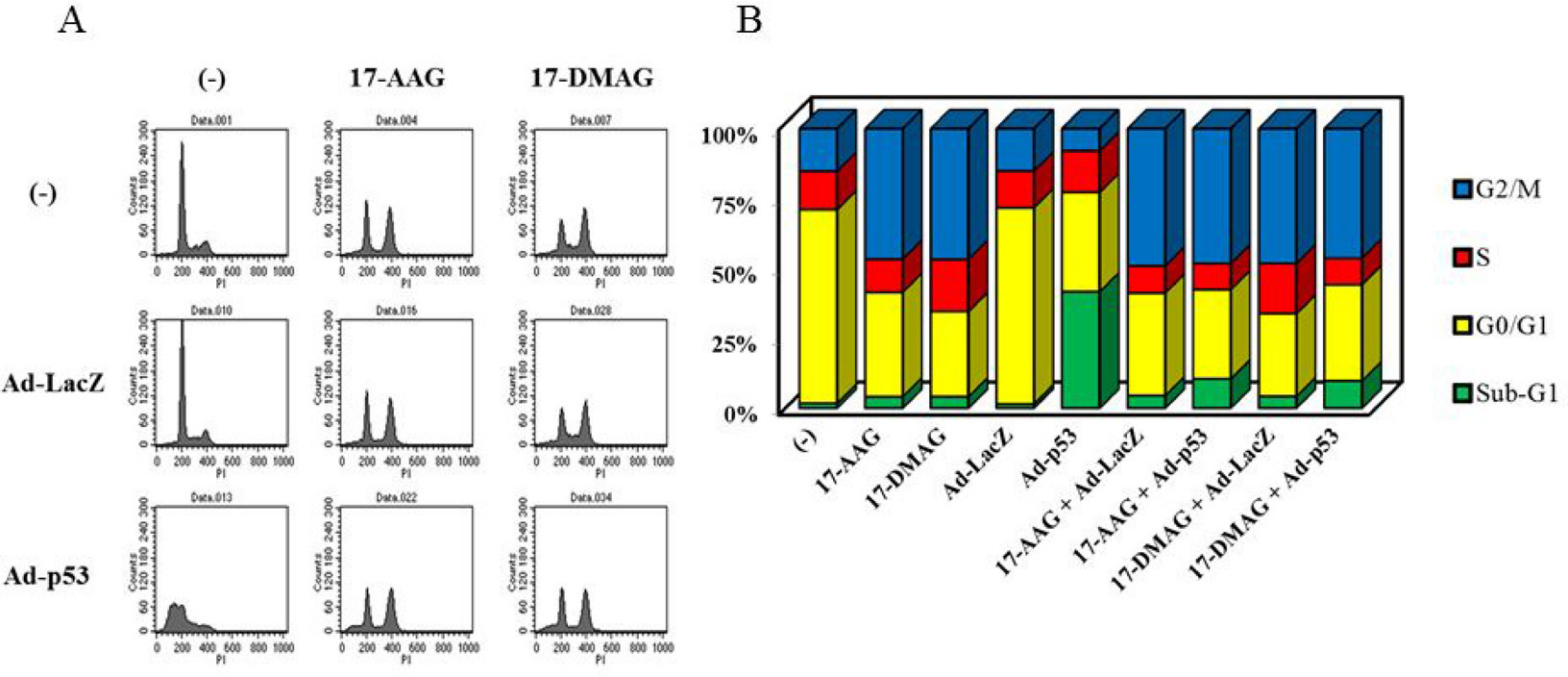
Cell cycle progression of JMN-1B cells infected with Ad-p53 and/or HSP90 inhibitors (**A**) Representative flow cytometry of JMN-1B cells which were untreated or treated with Ad-p53 (3 × 10^4^ vp/cell) or Ad-LacZ (3 × 10^4^ vp/cell) and with 17-AAG (1 μM) or 17-DMAG (0.1 μM) for 48 hours. (**B**) The data showed an average of respective subpopulations shown in Supplementary Table 3. The Y axis shows percentages of respective cell cycle populations.

We examined cell cycle profiles in JMN-1B cells treated with Ad-p53 and HSP90 inhibitors. Transduction of JMN-1B cells with Ad-p53 increased sub-G1 fractions but Ad-LacZ as a control did not influence the cell cycle (Figure 3, Supplementary Table 3). JMN-1B cells treated with the HSP90 inhibitors at that concentration increased G2/M phase populations with a little increase in sub-G1 fractions. Cells treated with combination of Ad-p53 and HSP90 inhibitors showed increased sub-G1 fractions but the sub-G1 increase was less than that in cells treated with Ad-p53 alone. The combination-treated cells rather increased G2/M populations as found in cells treated with HSP90 inhibitors. The flow cytometry thus indicated that HSP90 inhibitors suppressed Ad-p53-mediated effects on cell cycle progression.

We also examined influence of HSP90 inhibitors on Ad-p53-mediated cytotoxicity (Supplementary Figure 3). We previous demonstrated that transduction of mesothelioma with Ad-p53 inhibited cell growth and induced apoptosis [5]. Transduction of JMN-1B cells with Ad-p53 inhibited cell viability but combination with 17-AAG or 17-DMAG was rather inhibitory to Ad-p53-mediated cytotoxicity (Supplementary Figure 3A and 3B). Combination index (CI) values was above 1 in most of the fraction affected (Fa) points, which suggested that the combination produced antagonistic effects. We also examined NCI-H2452 cells expressing truncated p53 showed that Ad-p53 produced cytotoxic effects but additional 17-AAG was rather inhibitory to the Ad-p53-mediated effects (Supplementary Figure 3C). Furthermore, NCI-H2052 cells with the wild-type *p53* genotype was susceptible to Ad-p53-mediated inhibitory effects but combination with 17-AAG achieved antagonistic effects (Supplementary Figure 3D). These data indicated that HSP90 inhibitors decreased the Ad-p53-mediated effects through down-regulating p53 expression levels.

### Involvement of HSP70 in the down-regulated p53 expression

**Figure 4:**
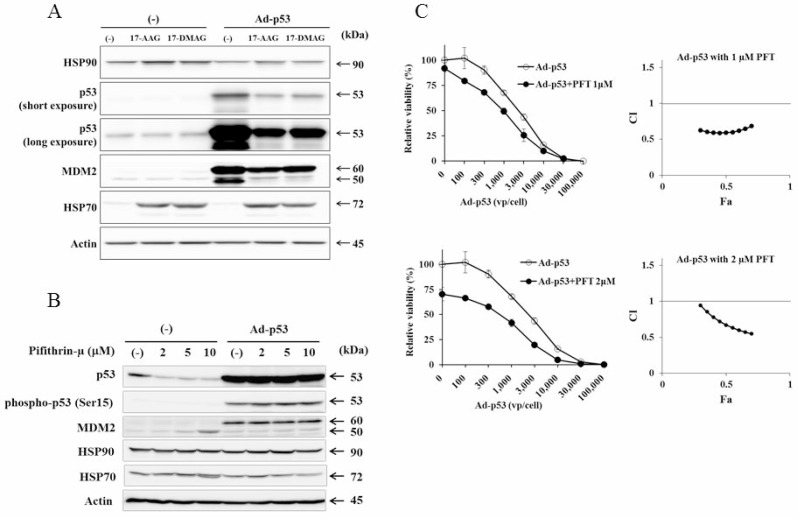
HSP70 was not involved in HSP90 inhibitors-mediated p53 suppression (**A**) Expression of HSP70 and HSP90 in JMN-1B cells that were untreated or treated with Ad-p53 (3 × 10^4^ vp/cell) and with 17-AAG (1 μM) or 17-DMAG (0.1 μM) for 48 hours was analyzed with Western blot analysis. Action was used as a loading control. (**B**) JMN-1B cells that were treated with Ad-p53 (3 × 10^4^ vp/cell) and/or PFT-μ as indicated for 48 hours were analyzed with Western blot analysis. Action was used as a loading control. (**C**) A combinatory use of Ad-p53 and PFT-μ in JMN-1B cells was tested with WST assay. The average with SE bars (*n* = 3) and CI values at Fa points between 0.3 and 0.7 are shown.

We investigated a possible involvement of HSP70 with a chaperone function in the down-regulated p53 expression by HSP90 inhibitors. JMN-1B cells treated with HSP90 inhibitors slightly increased HSP90 but markedly HSP70 levels irrespective of Ad-p53 infection (Figure 4A). We then examined influence of pifithrin-μ (PFT-μ), an HSP70 inhibitor, on the p53 levels induced by Ad-p53. PFT-μ suppressed expression of endogenous mutated p53 levels, but did not affect Ad-p53-induced p53 (Figure 4B). Expression of Ad-p53-mediated phosphorylated p53 and MDM2 was not influenced by PFT-μ although expression of truncated MDM2 at 50 kDa [12] was slightly augmented in uninfected cells. PFT-μ scarcely influenced HSP70 and HSP90 expression, but produced cytotoxicity to JMN-1B cells (Supplementary Figure 4). A combinatory use of Ad-p53 and PFT-μ did not show any antagonistic actions but rather produced synergistic cytotoxic activities (Figure 4C). These data collectively indicated that HSP70 was not involved in the down-regulated p53 expression induced by HSP90 inhibitors.

### Influence on Ad receptors' expression and infectivity of Ad by HSP90 inhibitors

**Figure 5:**
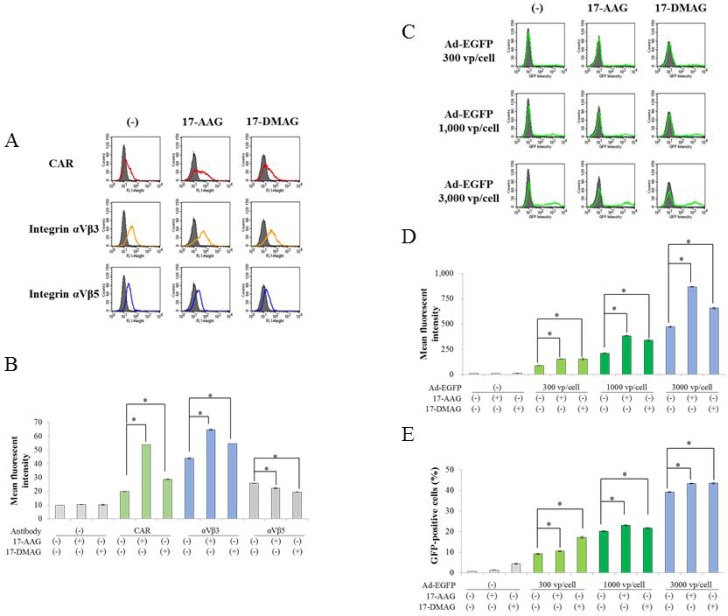
Influence of HSP90 inhibitors on Ad-mediated gene transfer (**A**) Representative flow cytometric profiles of CAR, integrin αVβ3 and αVβ5 molecules on JMN-1B cells which were untreated or treated with 17-AAG (1 μM) or 17-DMAG (0.1 μM) for 48 hours. Dark area in each histogram showed cells treated with secondary Ab alone. (**B**) Histograms indicating mean fluorescent intensity of JMN-1B cells treated with HSP90 inhibitors as above. SE bars were shown (*n* = 3). ^*^P < 0.01. (**C**) Infectivity and gene expression of Ad vector in HSP90 inhibitors-treated cells. JMN-1B cells were infected by various doses of Ad-EGFP for 2 hours, and then treated with 17-AAG (1 μM) or 17-DMAG (0.1 μM) for 48 hours. Intensity of GFP was analyzed with flow cytometry. Dark area showed uninfected cells. (**D**, **E**) Histograms indicating mean fluorescent intensity and GPF-positive percentages of JMN-1B cells treated as above. SE bars were shown (*n* = 3). ^*^P < 0.01.

We investigated a mechanism of down-regulation of Ad-p53-mediated p53 expression with HSP90 inhibitors. We firstly examined effects of HSP90 inhibitors on expression levels of Ad receptors and Ad infectivity (Figure 5). A major and subsidiary receptors of type 5 Ad were coxsackievirus and adenovirus receptor (CAR), and integrin αVb3 and αVβ5, respectively, and the cell surface receptor expression influenced the Ad-mediated gene expression. Flow cytometry showed that HSP90 inhibitors augmented expression levels of CAR and αVb3 but decreased that of αVβ5 in JMN-1B cells (Figure 5A and 5B). We then examined infectivity and gene expression by the Ad vector with the *enhanced green fluorescent protein* gene (Ad-EGFP) (Figure 5C–5E). JMN-1B cells infected with Ad-EGFP showed increased GFP intensity and GFP-positive cell numbers depending on Ad doses used, and HSP90 inhibitors augmented the intensity and increased the positive cell numbers. These data indicated that HSP90 inhibitors increased expression of the transduced gene. The down-regulation p53 with HSP90 inhibitors was thus not attributable to non-specific suppression of the transgene expression.

### HSP90 inhibitors suppressed p53 expression at a transcriptional and posttranscriptional level

**Figure 6:**
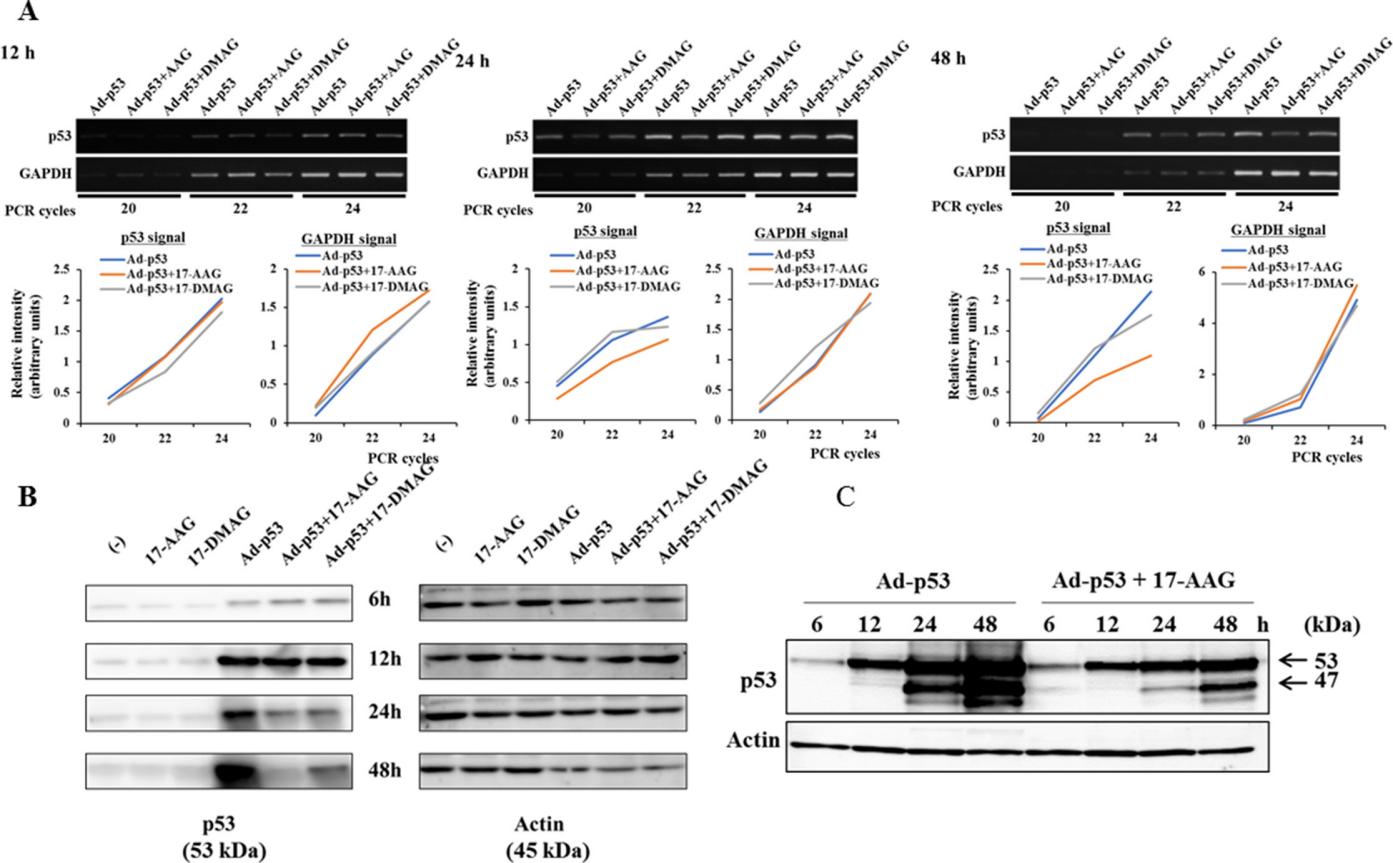
Influence of HSP90 inhibitor on p53 expression at the transcription and translation levels (**A**) Expression of p53 and GAPDH mRNA as a control was examined with semi-quantitative RT-PCR. JMN-1B cells infected with Ad-p53 (3 × 10^4^ vp/cell) and/or treated with 17-AAG (1 μM) or 17-DMAG (0.1 μM) were subjected to mRNA extraction at the time indicated and the RT-PCR was conducted with different PCR cycles as indicated. GAPDH expression was firstly adjusted and then p53 expression was tested. Relative intensity of PCR bands subtracted with background intensity was estimated with ImageJ software and expressed with arbitrary units. (**B**) Expression of p53 and actin as a control tested with Western blot analysis. JMN-1B cells treated as above were subjected to cell lysis at the time indicated. (**C**) JMN-1B cells were infected with Ad-p53 (3 × 10^4^ vp/cell) and/or treated with 17-AAG (1 μM) for the indicated time. Expression of p53 and actin as a control was analyzed with Western blot analysis.

We examined the suppressive activity of HSP90 inhibitors on p53 expression with reverse-transcription polymerase chain reaction (RT-PCR) and sequential Western blot analysis (Figure 6). The semi-quantitative RT-PCR analysis indicated that p53 transcripts of JMN-1B cells were not different at 12 hours among Ad-p53-infected cells untreated or treated with HSP90 inhibitors, but the amounts were marginally smaller in the inhibitor-treated cells than in untreated cells thereafter (Figure 6A). We tested p53 protein expression of JMN-1B cells at a different time point and found that the expression levels were almost similar up to 12 hours (Figure 6B). The p53 expression levels were lower in the inhibitor-treated cells than in untreated cells thereafter. Sequential p53 levels tested with the same Western blot showed that 17-AAG retarded increase of Ad-p53-mediated p53 expression (Figure 6C). These data showed that HSP90 inhibitors suppressed the p53 protein expression at a transcriptional and more significantly at a posttranscriptional level.

### Inhibited proteasome suppressed the down-regulated p53 expression

**Figure 7:**
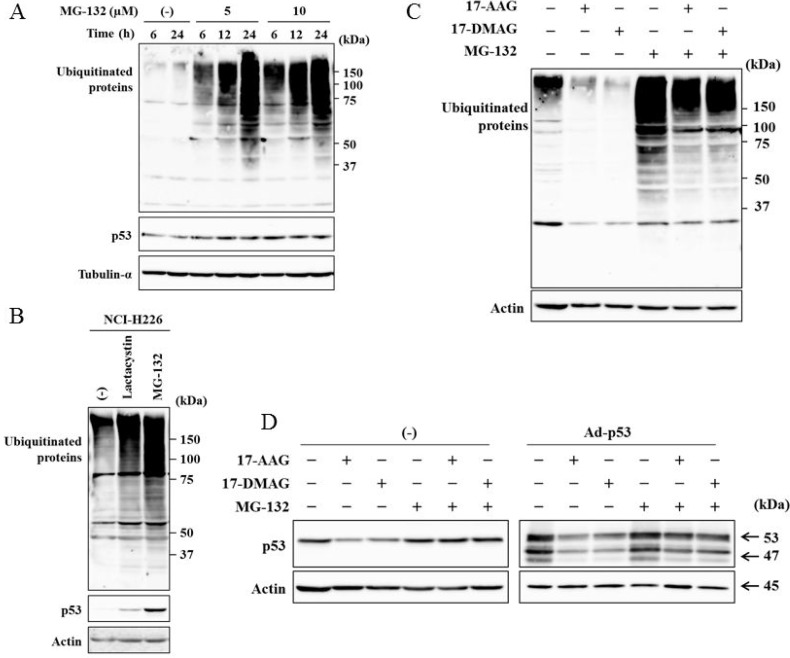
A proteasome inhibitor suppressed HSP90 inhibitor-mediated p53 down-regulation (**A**) JMN-1B or (**B**) NCI-H226 cells which were treated with MG-132(10 μM) or lactacystin (10 μM) were examined for ubiquitinated proteins and p53 expression with Western blot analysis. Tubulin-α and actin are used as a loading control. (**C**) JMN-1B cells were treated or untreated with MG-132 (10 μM) for 12 hours and then treated with 17-AAG (1 μM) or 17-DMAG (0.1 μM). The cell lysate was examined for ubiquitinated proteins and actin is used as a loading control. (**D**) JMN-1B cells uninfected or infected with Ad-p53 (3 × 10^4^ vp/cell) were treated or untreated with 17-AAG (1 μM), 17-DMAG (0.1 μM) and/or MG-132(10 μM) for 48 hours. The cell lysates were examined for p53 expression. Actin was used as loading control.

We investigated whether proteasome-mediated p53 degradation was involved in the p53 down-regulation by HSP90 inhibitors (Figure 7). HSP90 contributed to stabilization of a variety of proteins, and inhibition of HSP90 functions consequently lead to destabilization of the client proteins. We therefore treated JMN-1B cells with MG-132, a proteasome inhibitor, to examine a possible involvement of proteasome-mediated degradation. MG-132-treated JMN-1B cells accumulated ubiquitinated proteins due to inhibited proteasome activity and marginally increased mutate p53 expression (Figure 7A). NCI-H226 cells treated with MG-132 likewise increased endogenous wild-type p53 expression together with ubiquitinated proteins, and the increased p53 was also detected in cells treated with lactacystin, a proteasome inhibitor (Figure 7B). These data suggested that wild-type and to a less extent mutated p53 were subjected to proteasome-mediated degradation. We then treated JMN-1B cells with HSP90 inhibitors and MG-132 (Figure 7C). HSP90 inhibitors decreased ubiquitinated proteins in MG-132-untreated cells and also suppressed MG-132-enhanced ubiquitination, suggesting that HSP90 inhibitors stimulated protein degradation through ubiquitination-proteasome processes. We then examined p53 expression under MG-132 treatments (Figure 7D). MG-132 scarcely augmented endogenous p53 and exogenous p53 induced by Ad-p53 but blocked the p53 down-regulation by HSP90 inhibitors in Ad-p53-infected and uninfected cells (Figure 7D). These data collectively indicated that the HSP90 inhibitors-induced suppression of p53 expression was attributable to the proteasome-mediated degradation. Down-regulated expression of mutated p53 by HSP90 inhibitors suggested that mutated p53 was also degraded through proteasome-mediated degradation. Degradation of mutated p53 was however minor in comparison with wild-type p53 since the inhibitors did not suppress p53 expression in immunofluorescence analysis (Figure 2C, Supplementary Figure 2).

## DISCUSSION

In this study, we examined combinatory effects of Ad-p53 and HSP90 inhibitors on human mesothelioma cells and firstly showed an antagonistic action of HSP90 inhibitors on p53 expression induced with Ad-p53. The HSP90 inhibitors augmented endogenous wild-type p53 expression but decreased the exogenously expressed p53 levels. The present study therefore demonstrated a divalent function of HSP90 inhibitors regarding p53 expression.

HSP90 has a chaperon function to ensure correct conformation and stability of a number of client proteins. The inhibitors disrupt the biological roles and consequently induce cytotoxicity in cancer cells which are often dependent on the client proteins for their growth. The current study suggested that HSP90 inhibitors had 2 mechanisms regarding p53 stability. One is to augment endogenous wild-type p53 expression due to inhibition of MDM4 expression (Supplementary Figure 5). MDM4 constituted a complex with MDM2 bearing an ubiquitin ligase activity and promoted p53 degradation although MDM4 by itself did not have the ligase activity [13]. Inhibition of MDM4 functions can thus increase p53 levels and activate the p53 pathway. The current study showed that HSP90 inhibitors minimally suppressed p53 mRNA after 12 hours, while previous studies reported that the inhibitors did not influence the p53 transcript [14]. A functional role of HSP90 inhibitors in p53 transcription could therefore be minor but the inhibitors influenced p53 more significantly at the posttranslational level. HSP90 inhibitors increased p53 and the phosphorylation, but cytotoxicity of HSP90 inhibitors was not linked with the *p53* genotype or MDM4 expression levels, which suggested that the cytotoxicity was not directly dependent on augmented wild-type p53 expression. Nevertheless, HSP90 inhibitors increased sub-G1 fractions and cleaved PARP levels. We therefore presume that up-regulated p53 can play a certain role in the cell death although the inhibitors were more cytotoxic to cells of the non-p53 functional group. On the other hand, the inhibitors down-regulated expression of molecules associated with growth signals such as AKT (Supplementary Figure 5), which may contribute to the cytotoxicity irrelevant to p53. Augmentation of wild-type p53 increases sensitivity of tumor cells to DNA damaging anti-cancer agents, and the present study in fact showed that HSP90 inhibitors further increased p53 expression induced by cisplatin. These data suggests that a HSP90 inhibitor is a possible agent for mesothelioma therapy in combination of the current first-line agents since a majority of mesothelioma has the wild-type *p53* genotype. We also noticed that HSP90 inhibitors could decrease expression of endogenous mutated p53 in contrast to wild-type p53. MDM4 was therefore not involved in degradation of mutated p53 in mesothelioma.

**Figure 8:**
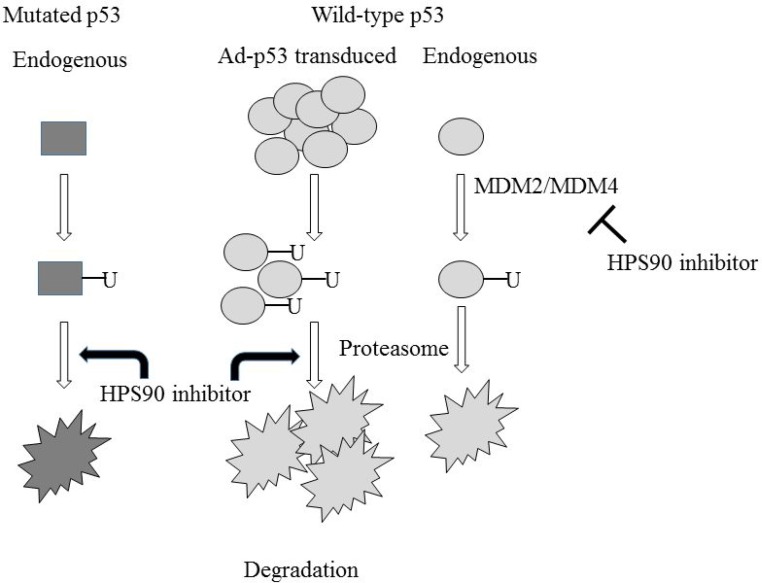
A working model of HSP90 inhibitors-mediated effects in p53 expression HSP90 inhibitors have a dual function for wild-type p53, inhibiting MDM2/MDM4-mediated ubiquitination to augment the expression and stimulating proteasome-mediated degradation to decrease the expression. Overproduction of p53 can further up-regulate the degradation. The inhibitors also facilitate proteasome-mediated degradation of mutated p53 but the effect on MDM2/MDM4-mediated ubiquitination of mutated p53 was unclear.

The other mechanism of HSP90 inhibitors on p53 expression clarified in the present study was facilitation of proteasome-mediated p53 degradation, which subsequently down-regulated adenovirally-transduced p53 expression. We demonstrated that an inhibitor for proteasome functions restored p53 expression which was suppressed by HSP90 inhibitors. We also showed that the proteasome inhibitor augmented expression of endogenous wild-type and less significantly mutated p53, and indicated that p53 molecules irrespective of the genotype were degraded through the proteasome pathway. HSP90 inhibitors facilitated the proteasome functions since the inhibitors decreased ubiquitinated protein levels even under MG-132 treatment. The present study showed that HSP90 inhibitors marginally decreased expression of mutated p53 and suppressed significantly Ad-p53-induced p53 levels, indicating that HSP90 inhibitors promoted ubiquitination-mediated p53 degradation regardless of the genotype. We presume that retarded increase of p53 levels in 17-AAG-treated cells with Ad-p53 transduction was attributable to the enhanced p53 degradation processes. Influences of HSP90 inhibitors demonstrated in the present study is summarized in Figure 8. Expression of endogenous wild-type p53 was augmented and that of overproduced wild-type p53 with Ad-p53 was suppressed by HSP90 inhibitors, whereas the inhibitors rather down-regulated endogenous mutated p53 through proteasome.

Previous studies showed that HSP90 inhibitors disrupted a stable complex of mutated p53 and MDM2, and mutated p53 released from the complex was subjected to degradation through ubiquitination [15, 16]. In contrast, an involvement of HSP90 in stability of wild-type p53 was controversial [17, 18]. Sasaki *et al*. even proposed that HSP90 inhibitors induced a conformational shift of wild-type p53 to a mutated form and decreased wild-type p53 levels through MDM2-depedent degradation of the mutated form [19]. These previous studies however did not investigate effects of HSP90 inhibitors in the case of p53 overproduction such as transduction with Ad-p53. A dual effect of HSP90 inhibitors on p53 expression, augmentation wild-type p53 and down-regulation of mutated p53, was previously reported [16, 20], but the present study firstly to our knowledge demonstrated the divalent effect on the same wild-type p53. It is currently unknown how differentially HSP90 inhibitors influenced wild-type p53 expression. The inhibitors augmented endogenous wild-type p53 by suppressing MDM4 expression and consequently blocking the MDM2-mediated p53 degradation. On the other hand, HSP90 directly functioned as a p53 chaperon in the p53-overproduced cells and the inhibitors suppressed the chaperon effects, which influenced the p53 levels greater than the inhibitory effect on MDM4. HPS90 inhibitors facilitated proteasome-mediated p53 degradation which exceeded the p53 augmentation effect when cells were under a p53-excess condition. Cisplatin-treated cells showed further augmentation of p53 with HSP90 inhibitors and presented a sharp contrast to Ad-p53-infected cells. It could be due to difference of p53 amounts produced. Cisplatin induced relatively low-leveled p53 expression compared with Ad-p53 and the HSP90-mediated chaperon effects could be minimal under the cisplatin-treated cells. The HSP90 inhibitors then showed the MDM4-inhibitory activity greater than blocking of the chaperon effects, and consequently p53 levels increased. A differential p53 induction system between cisplatin, which stimulated DNA damaging signals, and infection of a viral vector, can also be influential to divalent HSP90 functions. Cisplatin activated the p53 up-stream pathway and stabilized endogenous p53 thereafter, but infection with Ad vector directly produced p53 mRNA. Replication-incompetent Ad used in the present study may not induce DNA damages because Ad vector was not integrated into chromosome. The Ad may however induce a different type of cellular stress from DNA damages. A characteristic defect of p14 functions in mesothelioma may linked with the differential effects. The p14 defective state increased MDM2 levels and subsequently decreased a role of MDM4 in the p53 degradation since MDM2 by itself forms a homodimeric structure and the homodimer has a lower activity for p53 ubiquitination [21]. Moreover, p53 levels modulated by HSP90 inhibitors can be influenced by expression of co-chaperone molecules which form a complex with HSP90 [19].

HSP90 inhibitors augmented Ad vector-mediated EGFP gene expression which can be partly due to enhanced expression of Ad cellular receptors. The results showed that HSP90 inhibitors did not induce non-specific suppression of transgene expression, and consequently the p53 down-regulation was associated with p53 biosynthesis and the degradation. We also showed that the p53 down-regulation was not relevant to HSP70 molecules which served a similar chaperon function as HSP90 in terms of p53 expression [15, 22]. HSP90 inhibitors rather induced up-regulated HSP70 expression since decrease chaperon functions by the inhibitors were compensated by augmented HSP70. We showed that the Ad-p53-mediated p53 expression was not suppressed by PFT-μ. The agent is not a specific inhibitor for HSP70 but also blocks binding of p53 to mitochondria, and consequently inhibits apoptosis. The present study showed that PFT-μ inhibited mutated p53 expression and produced synergistic cytotoxicity with Ad-p53. These PFT-μ data were not concordant with those by HSP90 inhibitors and indicated that HSP70 was not involved in p53 down-regulation by HSP90 inhibitors. We also examine a possible involvement of mTOR and NF-κB pathways in the p53 suppression since HSP90 inhibitors influenced both pathways and p53 was also regulated by the pathways [23–25]. We treated JMN-1B cells with rapamycin, an inhibitor for mTOR functions, and infected with Ad-p53 (Supplementary Figure 6). JMN-1B cells treated with rapamycin showed dephosphorylation of p70S6 kinase, one of the targets of mTOR pathway, but rapamycin did not influence the p53 levels produced by Ad-p53. We then tested possible involvement of NF-κB activity in the down-regulated p53. We treated JMN-1B cells with BAY 11-7082, an inhibitor for IκBα degradation which consequenly suppressed the NF-κB pathway (Supplementary Figure 7). The inhibitor did not influence Ad-p53-mediated p53 expresion. These data showed that both pathways did not influence the p53 expression induced by Ad-p53. We however did not investigate p53 functions in terms of the tetrameric structure. Mutated p53 can bind to wild-type p53 to form the hetero-tetrameric structure, which were generated in JMN-1B cells infected with Ad-p53. Expression levels of such p53 complexes may be influenced by MDM4 and proteasome differently from those of p53 composed of wild-type or mutated type alone. The differential susceptibility of tetrameric p53 structure should be clarified in a future study.

A few studies reported anti-tumor effects of HSP90 inhibitors on mesothelioma [26, 27]. These previous reports analyzed the inhibitory actions on growth signal pathways but did not investigate a functional role of p53 in the cytotoxicity. Mesothelioma has a characteristic genetic alteration and restoration of the p53 pathway can be a therapeutic direction. An HSP90 inhibitor is one of the candidate agents for mesothelioma treatments but the divalent effect needs to be considered in any case of a combinatory use with other agents. The present study indicates that an inhibitor for chaperon functions can produce a possible dual effect to modulate expression of target molecules when these molecules and the relevant molecules are their client proteins.

## MATERIALS AND METHODS

### Cells

Human mesothelioma cells, MSTO-211H, NCI-H28, NCI-H226, NCI-H2052 and NCI-H2452, and immortalized mesothelial cells, Met-5A, were purchased from American Type Culture Collection (Manassas, VA, USA). Human mesothelioma, JMN-1B, EHMES-1 and EHMES-10 cells, which were established from Japanese patients (deceased), were kindly provided by Dr. Hironobu Hamada (Hiroshima University, Hiroshima, Japan) [28]. These cell lines were provided by an original establisher and an ethical approval is not required for the use. The genotype of *p53* was wild-type in MSTO-211H, NCI-H28, NCI-H226, NCI-H2052, EHMES-10 and NCI-H2452 cells, but p53 protein of NCI-H2452 cells was truncated (Supplementary Figure 1) [29]. In contrast, EHMES-1 (R273S) and JMN-1B (G245S) cells had mutated *p53*. All the *p53* wild-type mesothelioma cells have deficient expressions of p14^ARF^ and p16^INK4A^ resulting from either loss of the transcription or deletion of the genomic DNA. Met-5A cells, bearing the *p14^ARF^*, *p16^INK4A^* and wild-type *p53* genes, expressed SV40 T antigen and consequently p53 functions were inactivated. All the mesothelioma cells used in the present study were examined for the p53 status with single strand conformation polymorphism analysis and sequencing, and for the expression of p14 and p16 with genomic PCR and RT-PCR. Human pancreatic carcinoma AsPC-1 cells (American Type Culture Collection) with *p53*-null genotype were also used.

### Ad preparation

Replication-incompetent type 5 Ad-p53, Ad-LacZ and Ad-EGFP in which the cytomegalovirus promoter activated the transgenes, were prepared with an Adeno-X expression vector system (Takara, Shiga, Japan) and were purified with Adeno-X virus purification kit (BD Biosciences, Palo Alto, CA, USA). The number of virus particles (vp) per ml was estimated with the formula, absorbance at 260 nm in 0.1% sodium dodecyl sulfate × 1.1 × 10^12^.

### Cell viability test

Cells were seeded in 96-well plates (1 × 10^3^/well) and were treated with Ad, 17-AAG (Merck, St. Louis, MO, USA), 17-DMAG (Merck), or PFT- μ (2-phenylethynesulfonamide) (Merck). In a combinatory treatment, cells were infected with Ad-p53 or Ad-LacZ and then treated with 17-AAG, 17-DMAG or PFT- μ. They were cultured for 4 days and cell viability was determined with a cell-counting WST kit (Wako, Osaka, Japan) (WST assay) which detected formazan produced from WST-8 [2-(2-methoxy-4-nitrophenyl)-3-(4-nitrophenyl)- 5-(2,4-disulfophenyl)-2H-tetrazolium, monosodium salt] at 450 nm. The relative viability was calculated based on the absorbance without any treatments. IC_50_ and a CI at a Fa point which showed a relative suppression level of cell viability were calculated with the CalcuSyn software (Biosoft, Cambridge, UK). CI < 1, CI = 1 and CI > 1 indicate synergistic, additive and antagonistic actions, respectively.

### Western blot analysis

Cell lysate was subjected to sodium dodecyl sulfate polyacrylamide gel electrophoresis, transferred to a nitrocellulose membrane, and reacted with Ab against p53 (Ab-6, DO-1, Thermo Fisher, Cheshire, UK), phosphorylated p53 at Ser15 (#9284) or at Ser46 (#2521), caspase-8 (#9746), caspase-9 (#9502), poly (ADP-ribose) polymerase (PARP) (which also detects cleaved PARP) (#9542), LC3A/B (#4108), Beclin-1 (#3495), Atg5 (#2630), HSP90 (#4874), HSP70 (#4872), phosphorylated p70S6 kinase at Thr389 (#9205), IκBα (#9242), AKT (#9272), phosphorylated AKT at Ser 473 (#9271), p21 (#2947) (Cell Signaling, Danvers, MA, USA), MDM2 (sc-965, Santa Cruz Biotechnology, Santa Cruz, CA, USA), ubiquitin (ab7780, Abcam, Cambridge, MA, USA), MDM4 (A300-287A, Bethy Laboratories, Montogomery, TX, USA), actin (#4970) (Cell Signaling) and tubulin-α (MS-581-P1, Thermo Fisher) as a control. The membranes were exposured with the ECL system (GE Healthcare, Buckinghamshire, UK) and imaged with ImageQuant LAS 4000 (GE Healthcare).

### Cell cycle analysis

Cell were fixed with ethanol, treated with RNase (50 μg/ml) and stained with propidium iodide (PI) (50 μg/ml). The fluorescence intensity was analyzed with FACSCalibur (BD Biosciences) and CellQuest software (BD Biosciences).

### Immunofluorescence

Cells treated with Ad-p53 and/or HSP90 inhibitors were fixed with 4% para-formaldehyde, permeabilized with 0.2% Triton-X100 and then reacted with anti-p53 Ab, Pab1620 (OP33, EMD Millipore, Temecula, CA, USA) or DO-1 followed by incubation with Alexa Fluor 488-donkey anti-mouse IgG (R37114, Thermo Fisher Scientific, Waltham, MA) and were also counterstained with PI (50 μg/ml) to visualize the nuclei. They were then analyzed with Leica TCS SP8 with a 40× oil-immersion objective lens (Leica microsystems Inc., Deerfield, Illinois, USA).

### Flow cytometry

Cells were reacted with Ab against CAR (#05-644, Upstate, Lake Placid, NY, USA), integrin αVβ3 (MAB1976, Chemicon, Temecula, CA, USA) or αVβ5 (ab15459, Abcam), and followed by fluorescein isothiocyanate -conjugated goat anti-mouse IgG Ab (555748, BD Biosciences). They were then analyzed for the fluorescence intensity with FACSCalibur and CellQuest software.

### Infectivity of Ad

Cells were infected with Ad-EGFP with or without HSP90 inhibitors. Infected cells were cultured for 2 days and then analyzed for percentages of GFP-positive cells with FACSCalibur and CellQuest software. Cells of which fluorescence was greater than the brightest 5% of uninfected cells were judged as positively stained.

### RT-PCR

First-strand cDNA was synthesized with Superscript III reverse transcriptase (Invitrogen, Carlsbad, CA, USA) and amplification of equal amounts of the cDNA was performed with the following primers and conditions: for the *glyceraldehyde-3-phosphate dehydrogenase* (*GAPDH*) gene, 5′-ACCACAGTCCATGCCATCAC-3′ (sense) and 5′-TCCACCACCCTGTTGCTGTA-3′ (anti-sense), and 15 sec at 94°C for denature/15 sec at 60°C for annealing; for the *p53* gene, 5′-CTGCCCTCAACAAGATGTTTTG-3′ (sense) and 5′-CTATCTGAGCAGCGCTCATGG-3′ (anti-sense), and 30 sec at 96°C/90 sec at 65°C/28. PCR cycles were 20, 22 and 24 for both GAPDH and p53, and the intensity was measured after subtraction of a background level with ImageJ software (National Institute of Health, Bethesda, MD, USA, available at https://imagej.nihgov/ij/index.html).

### Statistical analysis

Experimental data were evaluated statistically with ANOVA test. A *P* value of less than 0.05 indicated statistical significance.

## SUPPLEMENTARY MATERIALS FIGURES AND TABLES





## Abbreviations

HSP90: heat shock protein 90
17-AAG: 17-allylamino-17-demetheoxygeldanamycin
17-DMAG: 17-dimethylaminoethylamino-17-demethoxy-geldanamycin
Ad-p53: adenoviruses expressing the wild-type p53 gene
Ad: adenoviruses
IC_50_: half maximal inhibitory concentration
Ad-LacZ: replication-incompetent type 5 Ad expressing the β-galactosidase
Ab: antibody
CI: combination index
Fa: fraction affected
PFT-μ: pifithrin-μ
CAR: coxsackievirus and adenovirus receptor
Ad-EGFP: replication-incompetent type 5 Ad expressing the *enhanced green fluorescent protein* gene
RT-PCR: reverse-transcription polymerase chain reaction
vp: virus particles
PI: propidium iodide
GAPDH: glyceraldehyde-3-phosphate dehydrogenase

## References

[1] Robinson BW, Musk AW, Lake RA (2005). Malignant mesothelioma. Lancet.

[2] Vogelzang NJ, Rusthoven JJ, Symanowski J, Denham C, Kaukel E, Ruffie P, Gatzemeier U, Boyer M, Emri S, Manegold C, Niyikiza C, Paoletti P (2003). Phase III study of pemetrexed in combination with cisplatin versus cisplatin alone in patients with malignant mesothelioma. J Clin Oncol.

[3] Lee AY, Raz DJ, He B, Jablons DM (2007). Update on the molecular biology of malignant mesothelioma. Cancer.

[4] Tagawa M, Tada Y, Shimada H, Hiroshima K (2013). Gene therapy for malignant mesothelioma: Current prospects and challenges. Cancer Gene Ther.

[5] Li Q, Kawamura K, Yamanaka M, Okamoto S, Yang S, Yamauchi S, Fukamachi T, Kobayashi H, Tada Y, Takiguchi Y, Tatsumi K, Shimada H, Hiroshima K (2012). Upregulated p53 expression activates apoptotic pathways in wild-type p53-bearing mesothelioma and enhances cytotoxicity of cisplatin and pemetrexed. Cancer Gene Ther.

[6] Trepel J, Mollapour M, Giaccone G, Neckers L (2010). Targeting the dynamic HSP90 complex in cancer. Nat Rev Cancer.

[7] Travers J, Sharp S, Workman P (2012). HSP90 inhibition: two-pronged exploitation of cancer dependencies. Drug Discov Today.

[8] Kim YS, Alarcon SV, Lee S, Lee MJ, Giaccone G, Neckers L, Trepel JB (2009). Update on Hsp90 inhibitors in clinical trial. Curr Top Med Chem.

[9] Wang X (2011). p53 regulation: teamwork between RING domains of Mdm2 and MdmX. Cell Cycle.

[10] Roh JL, Kim EH, Park HB, Park JY (2013). The Hsp90 inhibitor 17-(allylamino)-17-demethoxygeldanamycin increases cisplatin antitumor activity by inducing p53-mediated apoptosis in head and neck cancer. Cell Death Dis.

[11] Clegg HV, Itahana Y, Itahana K, Ramalingam S, Zhang Y (2012). Mdm2 RING mutation enhances p53 transcriptional activity and p53-p300 interaction. PLoS One.

[12] Pochampally R, Fodera B, Chen L, Shao W, Levine EA, Chen J (1998). A 60 kd MDM2 isoform is produced by caspase cleavage in non-apoptotic tumor cells. Oncogene.

[13] Herman AG, Hayano M, Poyurovsky MV, Shimada K, Skouta R, Prives C, Stockwell BR (2011). Discovery of Mdm2-MdmX E3 ligase inhibitors using a cell-based ubiquitination assay. Cancer Discov.

[14] Walerych D, Kudla G, Gutkowska M, Wawrzynow B, Muller L, King FW, Helwak A, Boros J, Zylicz A, Zylicz M (2004). Hsp90 chaperones wild-type p53 tumor suppressor protein. J Biol Chem.

[15] Peng Y, Chen L, Li C, Lu W, Chen J (2001). Inhibition of MDM2 by hsp90 contributes to mutant p53 stabilization. J Biol Chem.

[16] Li D, Marchenko ND, Schulz R, Fischer V, Velasco-Hernandez T, Talos F, Moll UM (2011). Functional inactivation of endogenous MDM2 and CHIP by HSP90 causes aberrant stabilization of mutant p53 in human cancer cells. Mol Cancer Res.

[17] Nagata Y, Anan T, Yoshida T, Mizukami T, Taya Y, Fujiwara T, Kato H, Saya H, Nakao M (1999). The stabilization mechanism of mutant-type p53 by impaired ubiquitination: the loss of wild-type p53 function and the hsp90 association. Oncogene.

[18] Müller L, Schaupp A, Walerych D, Wegele H, Buchner J (2004). Hsp90 regulates the activity of wild type p53 under physiological and elevated temperatures. J Biol Chem.

[19] Sasaki M, Nie L, Maki CG (2007). MDM2 binding induces a conformational change in p53 that is opposed by heat-shock protein 90 and precedes p53 proteasomal degradation. J Biol Chem.

[20] Lin K, Rockliffe N, Johnson GG, Sherrington PD, Pettitt AR (2008). Hsp90 inhibition has opposing effects on wild-type and mutant p53 and induces p21 expression and cytotoxicity irrespective of p53/ATM status in chronic lymphocytic leukaemia cells. Oncogene.

[21] Wade M, Wahl GM (2009). Targeting Mdm2 and Mdmx in cancer therapy: better living through medicinal chemistry?. Mol Cancer Res.

[22] Zylicz M, King FW, Wawrzynow A (2001). Hsp70 interactions with the p53 tumour suppressor protein. EMBO J.

[23] Ohji G, Hidayat S, Nakashima A, Tokunaga C, Oshiro N, Yoshino K, Yokono K, Kikkawa U, Yonezawa K (2006). Suppression of the mTOR-raptor signaling pathway by the inhibitor of heat shock protein 90 geldanamycin. J Biochem.

[24] Feng Z (2010). p53 regulation of the IGF-1/AKT/mTOR pathways and the endosomal compartment. Cold Spring Harb Perspect Biol.

[25] Rakitina TV, Vasilevskaya IA, O'Dwyer PJ (2003). Additive interaction of oxaliplatin and 17-allylamino-17-demethoxygeldanamycin in colon cancer cell lines results from inhibition of nuclear factor κB signaling. Cancer Res.

[26] Okamoto J, Mikami I, Tominaga Y, Kuchenbecker KM, Lin YC, Bravo DT, Clement G, Yagui-Beltran A, Ray MR, Koizumi K, He B, Jablons DM (2008). Inhibition of Hsp90 leads to cell cycle arrest and apoptosis in human malignant pleural mesothelioma. J Thorac Oncol.

[27] Ou WB, Hubert C, Corson JM, Bueno R, Flynn DL, Sugarbaker DJ, Fletcher JA (2011). Targeted inhibition of multiple receptor tyrosine kinases in mesothelioma. Neoplasia.

[28] Nakataki E, Yano S, Matsumori Y, Goto H, Kakiuchi S, Muguruma H, Bando Y, Uehara H, Hamada H, Kito K, Yokoyama A, Sone S (2006). Novel orthotopic implantation model of human malignant pleural mesothelioma (EHMES-10 cells) highly expressing vascular endothelial growth factor and its receptor. Cancer Sci.

[29] Di Marzo D, Forte IM, Indovina P, Di Gennaro E, Rizzo V, Giorgi F, Mattioli E, Iannuzzi CA, Budillon A, Giordano A, Pentimalli F (2014). Pharmacological targeting of p53 through RITA is an effective antitumoral strategy for malignant pleural mesothelioma. Cell Cycle.

