# Supplementary material for: Heat shock protein 90 inhibitors augment endogenous wild-type p53 expression but down-regulate the adenovirally-induced expression by inhibiting a proteasome activity
Source: Oncotarget. 2018 May 25;9(40):26130–43. doi: 10.18632/oncotarget.25452 (PMC5995238; doi:10.18632/oncotarget.25452)
Supplement: Supplementary file 1 [file oncotarget-09-26130-s001.pdf]

Heat shock protein 90 inhibitors augment endogenous wild-type p53 expression but down-regulate the adenovirally-induced expression by inhibiting a proteasome activity

SUPPLEMENTARY MATERIALS

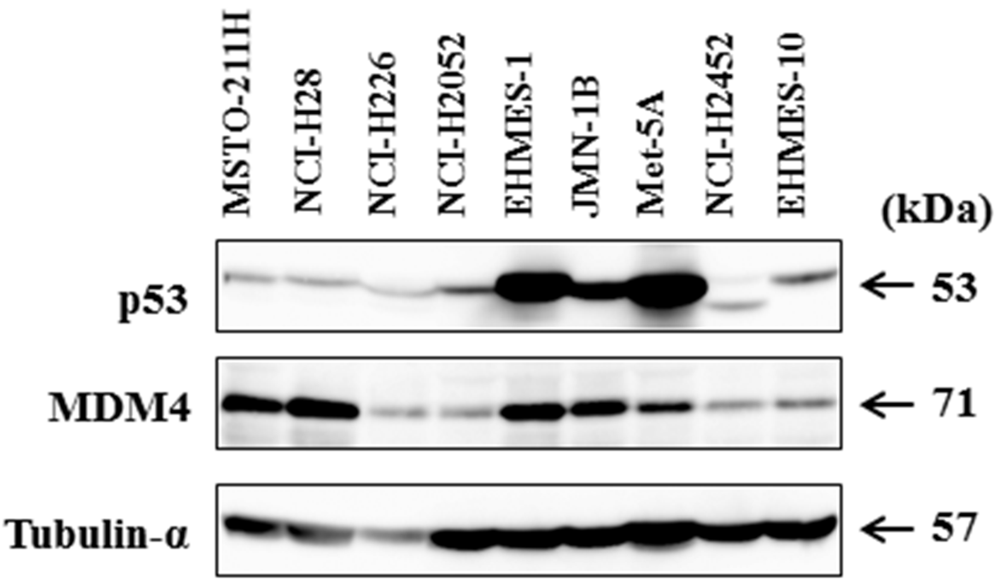

Supplementary Figure 1: Expression of p53 and MDM4 in mesothelioma examined with Western blot analysis. NCI-H2452 cells had truncated p53 protein and tubulin-α was used as a loading control.

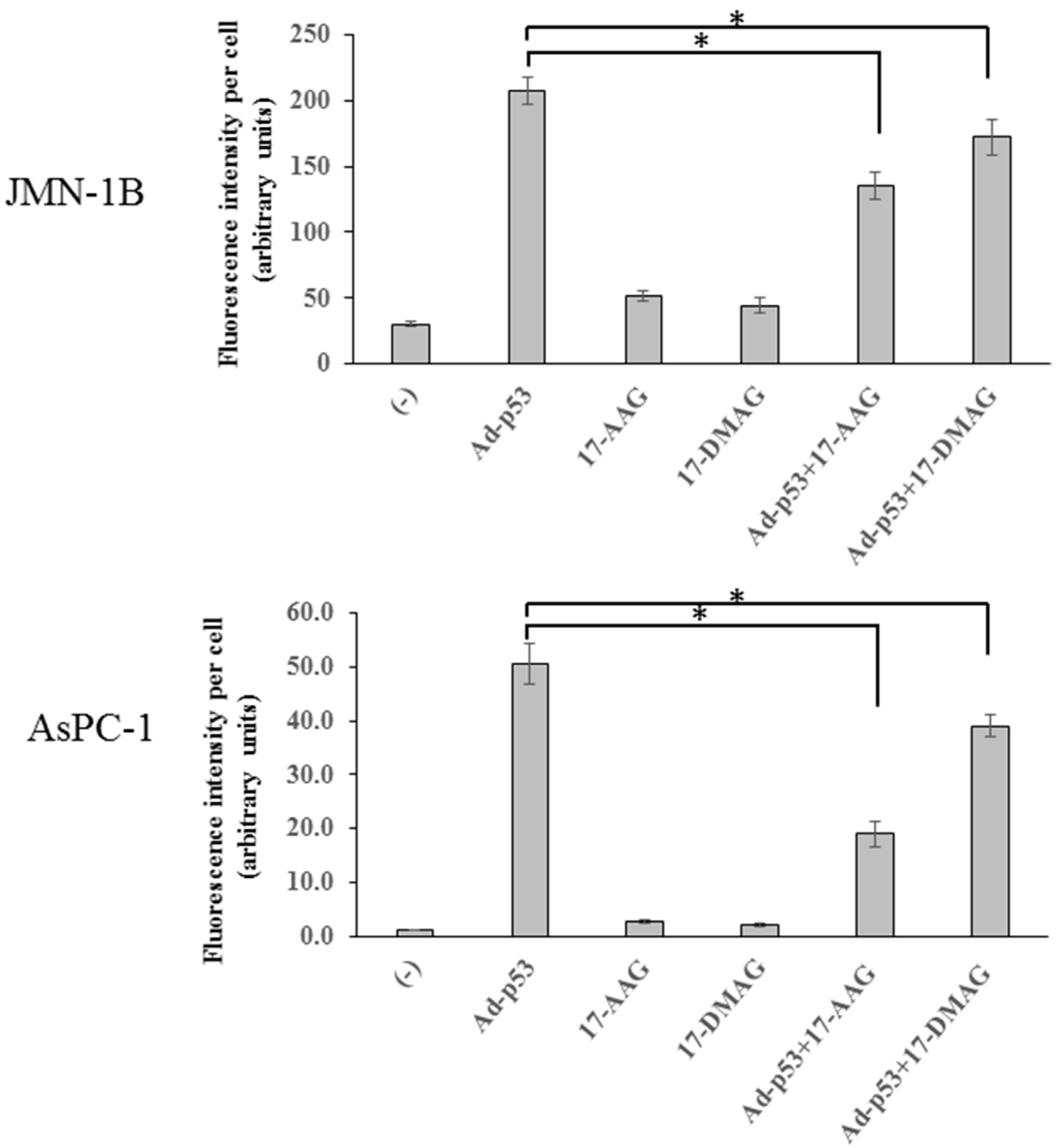

Supplementary Figure 2: HSP90 inhibitors decreased Ad-p53-induced p53 fluorescence intensity. JMN-1B or AsPC-1 cells treated with 17-AAG (1 μM) or 17-DMAG (0.1 μM), or with Ad-p53 (3×10^4 vp/cell) together with 17-AAG (1 μM) or 17-DMAG (0.1 μM), were stained with anti-p53Ab, DO-1 as shown in Figure 2C and 2F. The p53 fluorescence intensity of cells in a certain area after subtraction of the background (the size same area without stained cells) was measured with ImageJ software (National Institute of Health, Bethesda, MD, USA, available at https://imagej.nih gov/ij/index.html) and the intensity per cell was expressed as an arbitrary unit. $^{*}P < 0.01$ ($n = 6$, numbers of measured area).

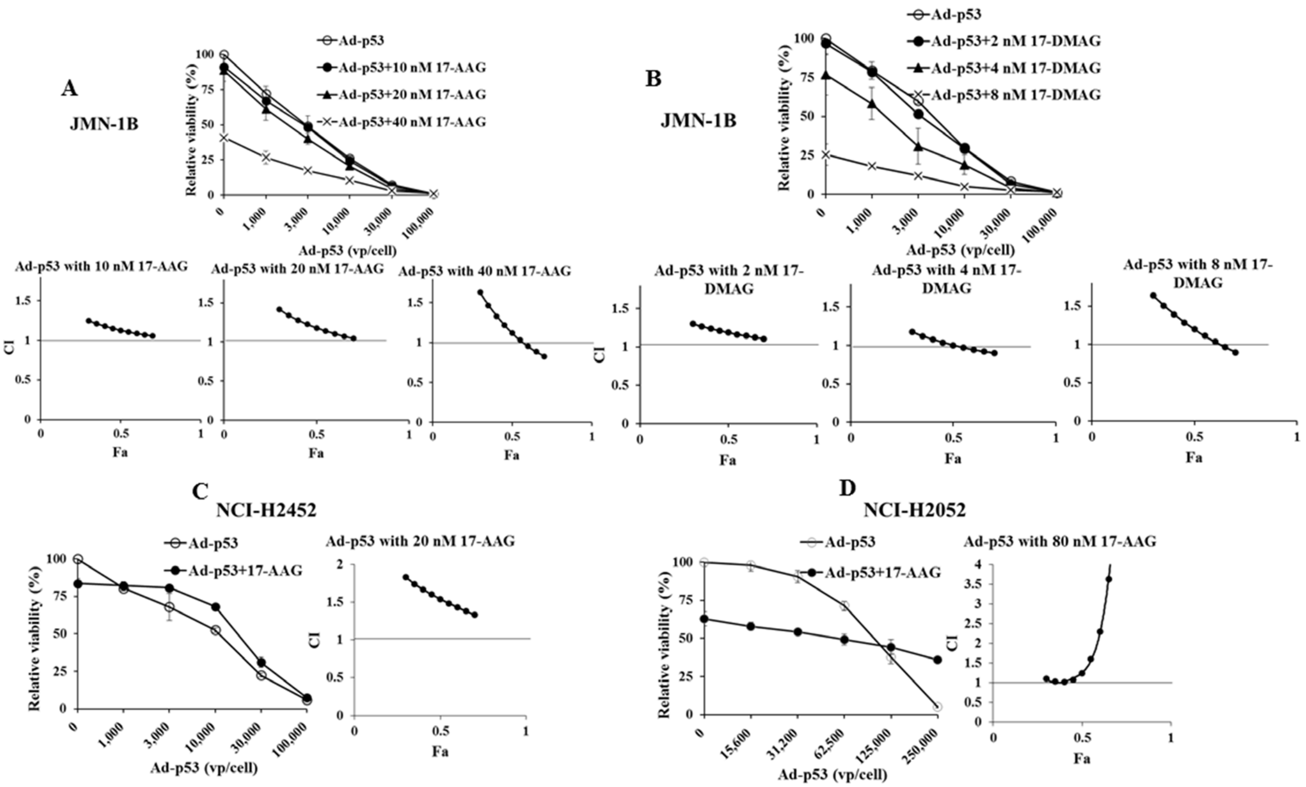

Supplementary Figure 3: Combinatory effects of Ad-p53 and HSP90 inhibitors. (**A, B**) JMN-1B, (**C**) NCI-H2452 and (**D**) NCI-H2052 cells were infected with Ad-p53 as indicated and treated with 17-AAG or 17-DMAG for 96 hours. Relative viability of cells was examined with the WST assay. The average with SE bars (*n* = 3) and CI values at Fa points between 0.3 and 0.7 are shown.

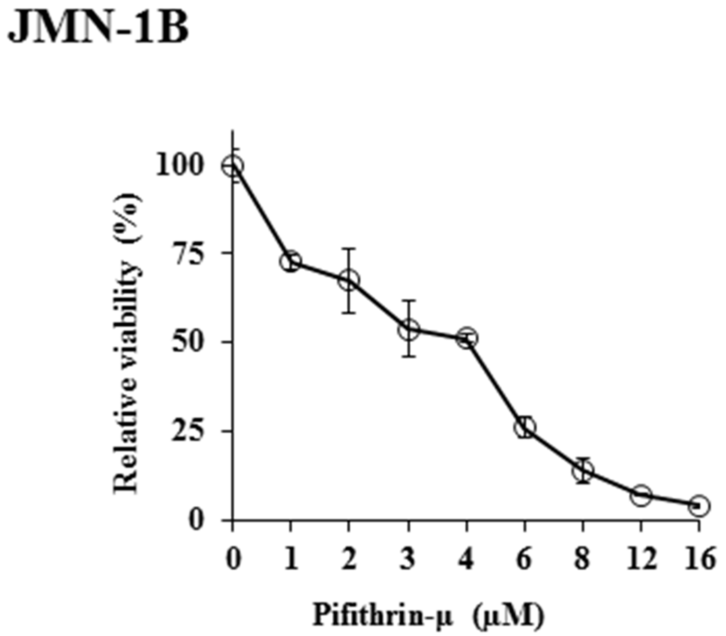

Supplementary Figure 4: Cytotoxicity of PFT-μ. JMN-1B cells were treated with various concentrations of PFT-μ and the cytotoxicity was examined with the WST assay. The average with SE bars (*n* = 3) are shown.

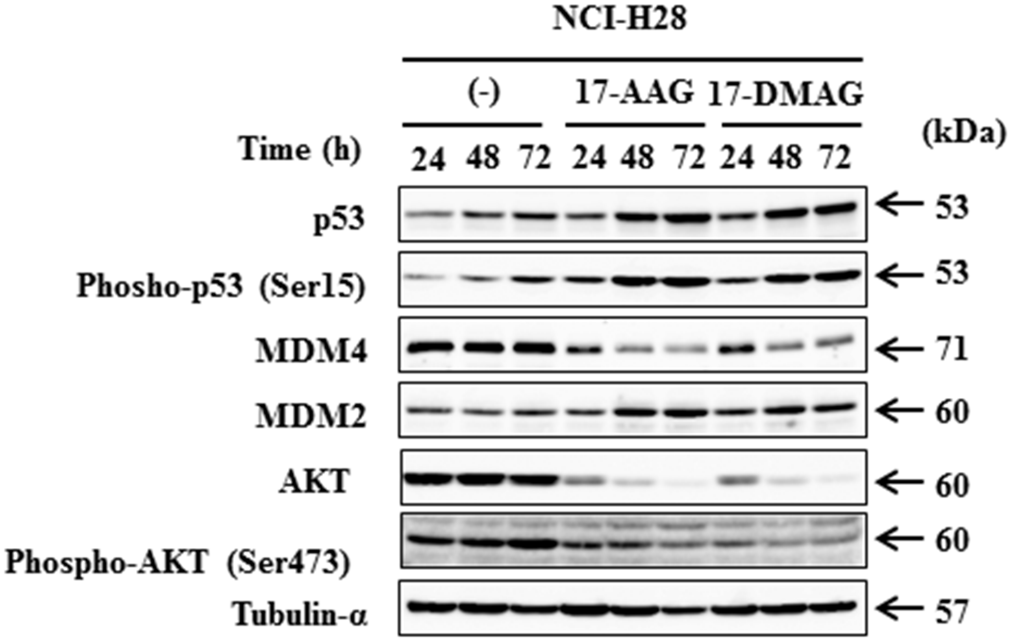

Supplementary Figure 5: Down-regulated expression of MDM4 with HSP90 inhibitors. NCI-H28 cells with wild-type *p53* genotype were treated with 17-AAG (0.3 μM) or 17-DMAG (0.1 μM) as indicated. Cell lysates were subjected to Western blot analysis. Tubulin-α was used as loading control.

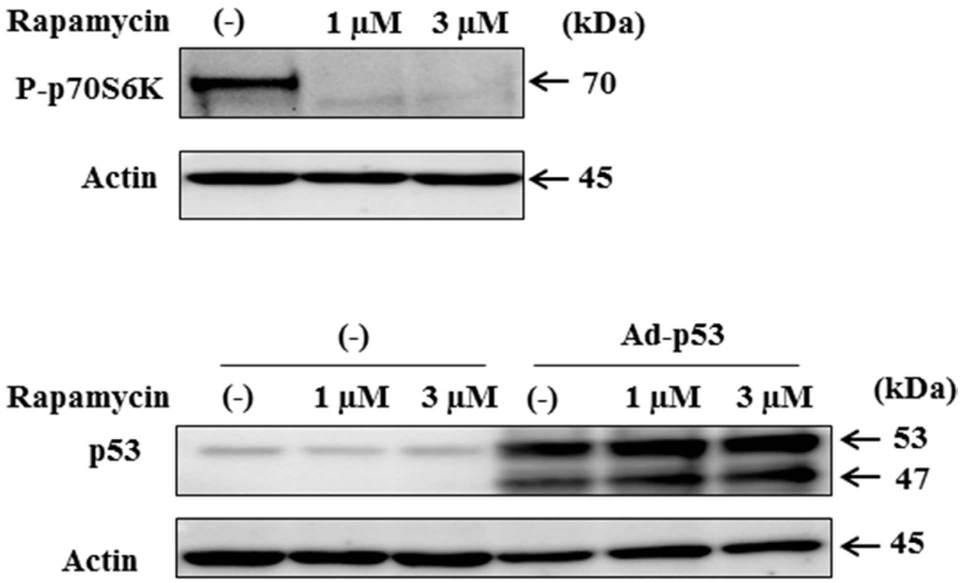

Supplementary Figure 6: A mTOR inhibitor did not influence HSP90 inhibitors-mediated p53 suppression. JMN-1B cells were uninfected or infected with Ad-p53 (3 × 10^4 vp/cell) and treated with rapamycin (1 or 3 μM) for 48 hours, and the cell lysate were subjected to Western blot analysis. Actin was used as a loading control.

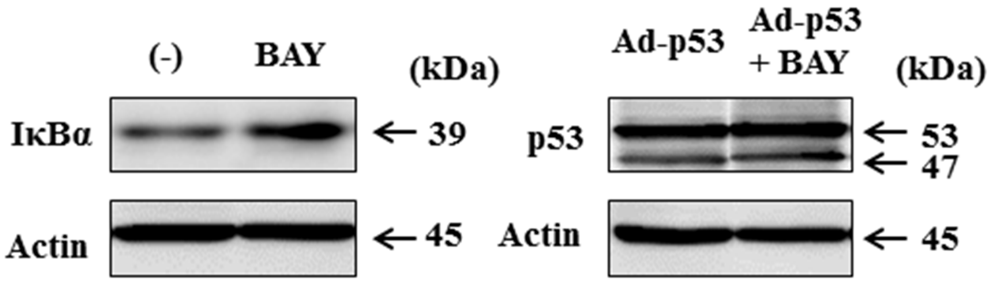

Supplementary Figure 7: Influence of the NF-*κB* pathway on p53 expression induced by Ad-p53. JMN-1B cells were treated with BAY 11-7082 (20 μM), an NF-κB inhibitor, and with Ad-p53 (3 × 10^4 vp/cell) for 48 hours. Cell lysates were subjected to Western blot analysis. Actin was used as loading control.

Supplementary Table 1: Signal intensity measured with ImageJ software. See Supplementary_Table_1

Supplementary Table 2: Cell cycle progression in MSTO-211H cells treated with HSP90 inhibitors

		Cell cycle distribution (%)			
**Agent**	**Time**	**Sub-G1**	**G0/G1**	**S**	**G2/M**
(-)	24	1.45 ± 0.05	62.18 ± 0.28	12.79 ± 0.19	23.97 ± 0.41
(-)	48	1.15 ± 0.07	67.26 ± 0.13	11.45 ± 0.24	20.46 ± 0.22
(-)	72	0.78 ± 0.05	72.28 ± 0.13	9.10 ± 0.24	18.09 ± 0.32
17-AAG	24	15.42 ± 0.32	42.45 ± 0.24	2.86 ± 0.06	39.64 ± 0.47
17-AAG	48	32.08 ± 0.55	36.18 ± 0.18	4.49 ± 0.14	27.38 ± 0.37
17-AAG	72	43.66 ± 0.96	31.42 ± 0.25	5.26 ± 0.18	19.78 ± 0.60
17-DMAG	24	10.27 ± 0.16	44.32 ± 0.45	2.37 ± 0.13	42.99 ± 0.41
17-DMAG	48	28.83 ± 0.37	35.62 ± 0.05	3.51 ± 0.06	32.10 ± 0.31
17-DMAG	72	46.67 ± 0.45	29.49 ± 0.27	5.32 ± 0.07	18.67 ± 0.55

MSTO-211H cells were treated with 17-AAG (2 µM) or 17-DMAG (1 µM) for 24, 48 or 72 hours. Data showed the average and SEs (n = 3).

Supplementary Table 3: Cell cycle progression in JMN-1B cells infected with Ad vector and/or the HSP90 inhibitors

Treatment		Cell cycle distribution (%)			
**Ad vector**	**Inhibitor**	**Sub-G1**	**G0/G1**	**S**	**G2/M**
(–)	(–)	1.74 ± 0.08	69.61 ± 0.20	13.84 ± 0.24	15.23 ± 0.10[a]
(–)	17-AAG	4.09 ± 0.20	37.62 ± 0.51	11.93 ± 0.23	46.90 ± 0.53[a]
(–)	17-DMAG	4.11 ± 0.09	30.70 ± 0.17	18.80 ± 0.27	47.03 ± 0.42[a]
Ad-LacZ	(–)	1.47 ± 0.01	70.45 ± 0.31	13.23 ± 0.17	15.12 ± 0.45
Ad-p53	(–)	42.01 ± 0.40[b]	35.86 ± 0.24	14.98 ± 0.28	7.98 ± 0.10
Ad-LacZ	17-AAG	4.46 ± 0.02	36.91 ± 0.20	9.75 ± 0.15	49.33 ± 0.15
Ad-LacZ	17-DMAG	4.22 ± 0.20	29.75 ± 0.15	18.07 ± 0.18	48.43 ± 0.47
Ad-p53	17-AAG	10.54 ± 0.40[b]	32.06 ± 0.33	9.35 ± 0.06	48.45 ± 0.48
Ad-p53	17-DMAG	9.83 ± 0.12[b]	34.55 ± 0.16	9.55 ± 0.06	46.53 ± 0.25

JMN-1B cells were treated with either 17-AAG (1 µM) or 17-DMAG (0.1 µM) and/or infected with Ad-p53 or Ad-LacZ (3×10^4 vp/cell) and cell cycle profiles at 48 hours were analyzed with flow cytometry. Data showed the average and SEs (n = 3).

[a]$P < 0.01$, comparing between cells treated with HSP90 inhibitors and untreated cells.

[b]$P < 0.01$, comparing between cells treated with combination of HSP90 inhibitors and Ad-p53 and those treated with Ad-p53 alone.
