# Supplementary material for: Heat shock protein 90 inhibitors augment endogenous wild-type p53 expression but down-regulate the adenovirally-induced expression by inhibiting a proteasome activity
Source: Oncotarget. 2018 May 25;9(40):26130–43. doi: 10.18632/oncotarget.25452 (PMC5995238; doi:10.18632/oncotarget.25452)
Supplement: Supplementary file 2 [file oncotarget-09-26130-s002.docx]

**Supplementary Table 1: Signal intensity measured with ImageJ software**

| Figure 1B  Phosphorylated p53 at Ser 15 | | | | | | | | | | |
| --- | --- | --- | --- | --- | --- | --- | --- | --- | --- | --- |
| Cells | (-) | DMSO | | | 17-AAG | | | 17-DMAG | | |
|  |  | 24 h | 48 h | 72 h | 24 h | 48 h | 72 h | 24 h | 48 h | 72 h |
| MSTO-211H | 0* | 0* | 0.3 | 0.8 | 2.6 | 4.6 | 4.5 | 2.2 | 6.5 | 8.0 |
| NCI-H28 | 0.3 | 1.0 | 2.0 | 3.7 | 4.3 | 4.9 | 4.0 | 4.8 | 4.5 | 1.3 |
| EHMES-10 | 0.1 | 0.2 | 0.3 | 0.5 | 4.3 | 4.5 | 2.5 | 5.9 | 4.7 | 2.4 |

| Figure 1C  Phosphorylated p53 at Ser 15 (long exposure) | | | | | |
| --- | --- | --- | --- | --- | --- |
| Untreated | | | CDDP treated | | |
| (-) | 17-AAG | 17-DMAG | (-) | 17-AAG | 17-DMAG |
| 1.7 | 3.7 | 4.1 | 26.2 | 31.0 | 32.7 |

| Figure 2A | | | | | | | | | |
| --- | --- | --- | --- | --- | --- | --- | --- | --- | --- |
| Molecules | (-) | 17-AAG | 17-DMAG | Ad-LacZ | Ad-p53 | 17-AAG + Ad-LacZ | 17-AAG + Ad-p53 | 17-DMAG + Ad-LacZ | 17-DMAG + Ad-p53 |
| p53 at 53 kDa | 2.4 | 2.0 | 2.8 | 1.6 | 16.8 | 2.2 | 6.8 | 1.6 | 7.0 |
| Phosphorylated p53 at Ser15 | 0* | 0.1 | 0.1 | 0* | 10.4 | 0.1 | 1.8 | 0.2 | 3.5 |
| p21 | 0* | 0.1 | 0* | 0* | 10.3 | 0.2 | 8.6 | 0* | 8.9 |
| Cleaved caspase-8 | 0* | 0* | 0* | 0* | 8.3 | 0* | 0* | 0* | 0.1 |
| Cleaved caspase-9 | 0.2 | 0.2 | 0.1 | 0* | 3.8 | 0* | 0* | *0 | 0.2 |
| LC3A/B I | 19.0 | 13.3 | 10.9 | 10.8 | 7.8 | 14.6 | 12.0 | 11.7 | 12.0 |
| LC3A/B II | 10.5 | 5.0 | 3.4 | 2.8 | 7.3 | 6.2 | 3.5 | 4.6 | 3.8 |

| Figure 2D | | | | | | | | | |
| --- | --- | --- | --- | --- | --- | --- | --- | --- | --- |
| Molecules | (-) | 17-AAG | 17-DMAG | Ad-LacZ | Ad-p53 | 17-AAG + Ad-LacZ | 17-AAG + Ad-p53 | 17-DMAG + Ad-LacZ | 17-DMAG + Ad-p53 |
| p53 at 53 kDa | 0* | 0* | 0* | 0.2 | 47.6 | 0* | 25.1 | 0* | 28.6 |
| p53 at 47 kDa | 0* | 0* | 0* | 0* | 58.7 | 0* | 3.8 | 0* | 4.5 |

| Figure 4A | | | | | | |
| --- | --- | --- | --- | --- | --- | --- |
| Molecules | (-) | | | Ad-p53 | | |
|  | (-) | 17-AAG | 17-DMAG | (-) | 17-AAG | 17-DMAG |
| HSP90 | 4.2 | 7.6 | 6.0 | 3.7 | 4.5 | 3.7 |
| p53 (short exposure) | 0* | 0* | 0* | 15.3 | 4.7 | 6.0 |
| HSP70 | 0.5 | 7.1 | 8.1 | 0.4 | 8.7 | 6.6 |

| Figure 4B: | | | | | | | | |
| --- | --- | --- | --- | --- | --- | --- | --- | --- |
| Molecules | (-) | | | | Ad-p53 | | | |
|  | Pifithrin-μ (µM) | | | | | | | |
|  | (-) | 2 | 5 | 10 | (-) | 2 | 5 | 10 |
| p53 | 4.8 | 1.4 | 1.7 | 1.8 | 14.1 | 16.3 | 17.0 | 17.3 |
| Phosphorylated p53 at Ser15 | 0.1 | 0.1 | 0.1 | 0.2 | 3.7 | 4.7 | 5.1 | 5.0 |

| Figure 6B  p53 protein (Adjusted by actin intensity) | | | | | | |
| --- | --- | --- | --- | --- | --- | --- |
| Time (hours) | (-) (adjusted to 1.0) | 17-AAG | 17-DMAG | Ad-p53 | Ad-p53 + 17-AAG | Ad-p53 + 17-DMAG |
| 6 | 1.0 | 0.9 | 0.5 | 3.6 | 7.1 | 6.8 |
| 12 | 1.0 | 0.7 | 1.9 | 18.1 | 14 | 9.4 |
| 24 | 1.0 | 1.0 | 1.6 | 17.4 | 7.8 | 10.0 |
| 48 | 1.0 | 1.1 | 2.0 | 22.9 | 8.6 | 12.9 |

| Figure 6C  p53 protein | | | | | | | | |
| --- | --- | --- | --- | --- | --- | --- | --- | --- |
|  | Ad-p53 | | | | Ad-p53+17-AAG | | | |
| Time (hours) | 6 | 12 | 24 | 48 | 6 | 12 | 24 | 48 |
| p53 at 53 kDa | 4.4 | 20.5 | 38.2 | 47.1 | 10.3 | 17.3 | 22.6 | 28.7 |
| p53 at 47 kDa | 0* | 2.6 | 25.0 | 39.2 | 3.1 | 1.2 | 6.5 | 20.4 |

| Figure 7A | | | | | | | | | |
| --- | --- | --- | --- | --- | --- | --- | --- | --- | --- |
| Molecules |  | MG-132 (μM) | | | | | | | |
|  | Concentrations | (-) | | 5 | | | 10 | | |
|  | Time (hours) | 6 | 24 | 6 | 12 | 24 | 6 | 12 | 24 |
| Ubiquitinated proteins |  | 3.3 | 5.7 | 41.7 | 70.1 | 122.2 | 62.1 | 77.8 | 105.4 |
| p53 |  | 2.3 | 1.6 | 3.1 | 3.7 | 3.4 | 4.6 | 3.3 | 3.4 |

| Figure 7B | | | |
| --- | --- | --- | --- |
| Molecules | (-) | Lactacystin | MG-132 |
| Ubiquitinated proteins | 66.9 | 117.5 | 141.7 |
| p53 | 0.1 | 1.0 | 4.7 |

| Figure 7C | | | | | | | |
| --- | --- | --- | --- | --- | --- | --- | --- |
| Molecules | 17-AAG | - | + | - | - | + | - |
|  | 17-DMAG | - | - | + | - | - | + |
|  | MG-132 | - | - | - | + | + | + |
| Ubiquitinated proteins | | 60.9 | 13.7 | 9.8 | 190 | 113.4 | 118.2 |

| Figure 7D | | | | | | | | | | | | | |
| --- | --- | --- | --- | --- | --- | --- | --- | --- | --- | --- | --- | --- | --- |
| Molecules |  | (-) | | | | | | Ad-p53 | | | | | |
|  | 17-AAG | - | + | - | - | + | - | - | + | - | - | + | - |
|  | 17-DMAG | - | - | + | - | - | + | - | - | + | - | - | + |
|  | MG-132 | - | - | - | + | + | + | - | - | - | + | + | + |
| p53 at 53 kDa | | 7.7 | 3.0 | 4.3 | 7.6 | 7.9 | 8.1 | 13.5 | 6.0 | 8.1 | 14.7 | 11.2 | 11.7 |
| p53 at 47 kDa | | 0* | 0* | 0* | 0* | 0* | 0* | 12.8 | 4.2 | 4.7 | 13 | 7.3 | 9.0 |

| Supplementary Figure 5 | | | | | | | | | | |
| --- | --- | --- | --- | --- | --- | --- | --- | --- | --- | --- |
|  | (-) | | | 17-AAG | | | 17-DMAG | | | |
| Time (hours) | 24 | 48 | 72 | 24 | 48 | 72 | 24 | 48 | | 72 |
| Phosphorylated p53 a Ser15 | 1.1 | 1.7 | 3.5 | 3.3 | 5.9 | 6.5 | 2.9 | 5.4 | 5.9 | |
| MDM4 | 6.8 | 6.0 | 6.7 | 3.5 | 2.1 | 2.0 | 4.4 | 2.3 | 2.8 | |
| MDM2 | 3.0 | 2.5 | 3.2 | 2.7 | 5.1 | 5.3 | 3.5 | 4.8 | 4.5 | |
| AKT | 7.8 | 7.8 | 7.3 | 2.6 | 1.0 | 0.3 | 2.7 | 0.8 | 0.4 | |
| Phosphorylated AKT at Ser473 | 3.9 | 4.2 | 5.8 | 2.9 | 3.0 | 1.8 | 1.8 | 0.9 | 1.1 | |

| Supplementary Figure 6 upper panel | | | |
| --- | --- | --- | --- |
| Molecules | Rapamycin (µM) | | |
|  | (-) | 1 | 3 |
| Phosphorylated p70S6K | 30.1 | 2.9 | 1.8 |

| Supplementary Figure 6 lower panel | | | | | | |
| --- | --- | --- | --- | --- | --- | --- |
|  | (-) | | | Ad-p53 | | |
|  | Rapamycin (µM) | | | | | |
| Concentrations | (-) | 1 | 3 | (-) | 1 | 3 |
| p53 at 53 kDa | 4.3 | 3.0 | 4.6 | 31.6 | 44.2 | 43.3 |
| p53 at 47 kDa | 0.9 | 0.3 | 1.2 | 24.0 | 30.8 | 35.3 |

| Supplementary Figure 7 | | |
| --- | --- | --- |
| Molecules | (-) | BAY |
| IκBα | 16.2 | 26.9 |

| Supplementary Figure 8 | | |
| --- | --- | --- |
| Molecules | Ad-p53 | Ad-p53 + BAY |
| p53 at 53 kDa | 25.7 | 29.8 |
| p53 at 47 kDa | 12.3 | 15.4 |

Intensity of chemiluminescence was measured after subtraction of a background level with ImageJ software (National Institute of Health, Bethesda, MD, USA, available at <https://imagej.nih> gov/ij/index.html). Intensity is shown as an arbitrary unit standardized by control intensity (actin and GAPDH or tubulin-α).

0*: undetectable
